# K^+^-Cl^−^ cotransporter 1 (KCC1): a housekeeping membrane protein that plays key supplemental roles in hematopoietic and cancer cells

**DOI:** 10.1186/s13045-019-0766-x

**Published:** 2019-07-11

**Authors:** A. P. Garneau, S. Slimani, L. E. Tremblay, M. J. Fiola, A. A. Marcoux, P. Isenring

**Affiliations:** 1 From the Nephrology Research Group, Department of Medicine, Laval University, 11, côte du Palais, Québec (Qc), G1R 2J6 Canada; 2 Cardiometabolic Axis, School of Kinesiology and Physical Activity Sciences, University of Montréal, 900, rue Saint-Denis, Montréal (Qc), H2X 0A9 Canada; 3 L’Hôtel-Dieu de Québec Institution, 10, rue McMahon, Québec (Qc), G1R 2J6 Canada

**Keywords:** Cation-Cl^−^ cotransporter, K^+^-Cl^−^ cotransporter, Red blood cells, Sickle cell anemia, Abnormal cell growth, Animal models, Submitted to Journal of Hematology and Oncology

## Abstract

During the 1970s, a Na^+^-independent, ouabain-insensitive, N-ethylmaleimide-stimulated K^+^-Cl^−^ cotransport mechanism was identified in red blood cells for the first time and in a variety of cell types afterward. During and just after the mid-1990s, three closely related isoforms were shown to account for this mechanism. They were termed K^+^-Cl^−^ cotransporter 1 (KCC1), KCC3, and KCC4 according to the nomenclature of Gillen et al. (1996) who had been the first research group to uncover the molecular identity of a KCC, that is, of KCC1 in rabbit kidney. Since then, KCC1 has been found to be the most widely distributed KCC isoform and considered to act as a housekeeping membrane protein. It has perhaps received less attention than the other isoforms for this reason, but as will be discussed in the following review, there is probably more to KCC1 than meets the eye. In particular, the so-called housekeeping gene also appears to play crucial and specific roles in normal as well as pathological hematopoietic and in cancer cells.

## Introduction

K^+^-Cl^−^ cotransporter 1 (KCC1) is a membrane protein that mediates the symport of K^+^ and Cl^−^ ions through the surface of most animal cells [1]. It is also referred to as SLC12A4 based on the Human Genome Organization (HUGO) nomenclature. It shares high levels of homology in amino acid sequence with three other KCC isoforms that are termed KCC2 (SLC12A5), KCC3 (SLC12A6), and KCC4 (SLC12A7). KCC1 also shares moderate levels of homology with three additional proteins that mediate the symport movement of Na^+^ and Cl^−^ in the absence or presence of K^+^. Along with the four KCC isoforms, these additional proteins are all part of a larger family of proteins that are termed cation-Cl^−^ cotransporters (CCC) in the literature [1–4].

The molecular identity of KCC1 was deciphered during the mid-1990s just after that of the Na^+^-dependent CCC. Of notice, however, pioneer work by three research groups had already led to the identification of a K^+^-Cl^−^ cotransport mechanism during the seventies [5–7]. Subsequent to their discoveries, the KCC were eventually found to exhibit unique physiological roles and distribution patterns. KCC2 and KCC3 have received the most attention as they were ultimately linked to hereditary forms of neurological disorders in human [8–10]. KCC1 has received much less attention given that it was found to be ubiquitously distributed and assumed to act as a housekeeping gene [11–13].

There are yet many lines of evidence to suggest that KCC1 accomplishes dedicated physiological roles as well. In particular, this isoform has been shown to sustain normal erythropoiesis, sickle cell formation, cancer growth, and bone turnover [14–18]. The main goal of the following review will be to discuss the molecular features and tissue-specific functions of KCC1 from the hematological perspective for the greater part. As will be seen, the characterization of KCC1 has led the way to important findings and promising therapeutic avenues.

## Main text

### Identification of KCC1, a member of the cation-Cl^−^ cotransporter family

#### Early functional characterization

In the seventies, a Na^+^-independent K^+^-Cl^−^ cotransport mechanism was formally identified for the first time in red blood cells (RBC) by three research groups [5–7]. It was found to exhibit saturation kinetics and to be stimulated by cell swelling as well as N-ethylmaleimide, a thiol-reacting agent. It was also suspected of allowing reticulocytes (RTC) to decrease their cell volume while maturing into erythrocytes.

Soon after this discovery, several tissues and cell types were found to express a K^+^-Cl^−^ cotransport mechanism that was more active under the hypotonic condition and that differed from another mechanism known as Na^+^-K^+^-Cl^−^ cotransport. They included mouse ascites tumor cells [19], bovine aortic endothelial cells [20], salamander gallbladder, and proximal nephron [21, 22] as well as many other cell types or tissues.

Further studies eventually showed that the K^+^-Cl^−^ cotransport mechanism was inhibited by the loop diuretic furosemide and the alkanoic acid DIOA [11, 23, 24]. They also shed light on the mechanisms by which this transport moiety is regulated in response to cell swelling. Such mechanisms were found to involve the cytoskeleton [25, 26] and signaling intermediates that cause the carrier to undergo dephosphorylation [27, 28].

#### Initial molecular characterization

A protein responsible for K^+^-Cl^−^ cotransport was uncovered for the first time in rabbit kidney and rat brain during the mid-nineties, that is, almost 20 years after the initial functional characterizations in RBC. It was termed K^+^-Cl^−^ cotransporter 1 (KCC1) by the research group who had made the discovery [11]. Another isoform (KCC2) was uncovered during the same time [29] and two other isoforms (KCC3 and KCC4) a few years later [30–32].

To clone KCC1, the strategy used was based on the observation that K^+^-Cl^−^ and Na^+^-K^+^-Cl^−^ cotransport shared various functional traits [33] and that the proteins responsible for either mechanism would thus share homology in residue sequences as well. Because the Na^+^-K^+^-Cl^−^ cotransporters (NKCC) had already been cloned through previous work, they would then serve as queries to identify a putative KCC among orphan expressed sequence tags (EST) [2, 34, 35].

**Fig. 1:**
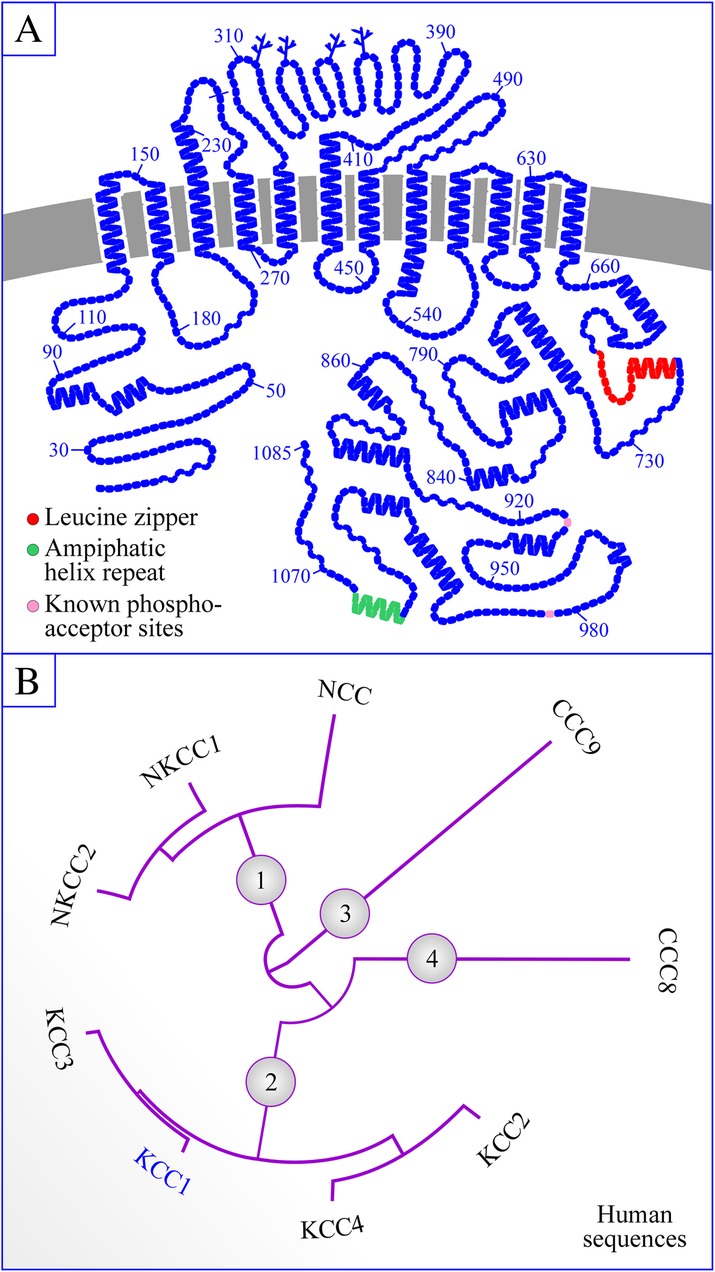
Structure of KCC1 and classification of the CCC family. a Structure. The topology model shown was drawn with the program PLOT by Biff Forbush (Yale University). Branched lines correspond to glycosylation sites, other symbols to residues and colors other than blue, to functional sites of potential importance. b Phylogenetic tree of the CCC family. The phylogram shown was obtained with the programs PhyML v3.1/3.0 aLRT and MUSCLE v3.8.31 [36, 37] using the most abundant human variants. GenBank accession numbers are provided in footnote 1^1^

The strategy exploited led to the identification of EST that shared 20–50% homology with the NKCC sequences and encompassed the 3′ end of a candidate transporter [11]. A rabbit kidney medulla cDNA library was subsequently screened with one of the EST identified and found to include a large sequence that was comprised of a 3255-bp full-length open reading frame. This open reading frame was eventually predicted to encode a 12-transmembrane domain glycoprotein that shared moderate levels of homology in amino acid sequence with the NKCC (Fig. 1a).

Studies in rabbit KCC1-expressing HEK-293 cells confirmed that the clone identified encoded a K^+^-Cl^−^ cotransport mechanism [11]. In the presence of N-ethylmaleimide, for instance, the initial rate of furosemide-sensitive Rb^+^ efflux from these cells was 8-fold higher compared to controls cells. Along the same line, Rb^+^ efflux was stimulated further through cell swelling and Rb^+^ uptake by KCC1 was Cl^−^-dependent as well as Na^+^-independent.

#### Splice variants

KCC1 is expressed as four splice variants in the mouse as well as in human tissues. However, KCC1A is the only transcript to be fully conserved between the two species given that it is produced through the same initiation site in exon 1A and includes the same 24 exons. KCC1A is also the only transcript for which a role has been clearly defined. The other transcripts are formed through the alternative usage of three exons (called 1A, 1B, or 1C) in human and of two initiation sites along exon 1A in human and mouse. Some of the other transcripts also lack an exon in part or in full and one of the transcripts in mouse lacks most of the open reading frame.

#### Cation-Cl^−^ cotransporter family

As it stands, nine CCC family members are known to exist. They fall into different phylogenetic branches as follows (and as shown in the cladogram of Fig. 1): KCC1 belongs to one branch along with the three other KCC, the Na^+^-dependent CCC belong to another branch and the remaining two CCC (called CCC8 and CCC9) belong to each an independent branch. Overall, KCC1 shares higher identity with KCC3 (~ 75%) than with any of the other CCC [1, 3].

### Localization of KCC1 in animal species

#### Tissue distribution

Northern blot analyses of rat tissues initially revealed that KCC1 was ubiquitously expressed [11]. These observations are still supported today by the presence of KCC1 in numerous cell types and among the current EST sequences of multiple tissues from human, mouse, and rat. For this reason and due to its transport function, *Kcc1* is often considered as a housekeeping gene that is involved in cell volume regulation and intracellular electrolyte balance [11–13].

As for the erythroid and lymphoid systems more specifically, KCC1 has been detected in bone marrow, lymph nodes, spleen, macrophages, pluripotent stem cells, RTC, mature RBC, megakaryocytes, circulating T cells, and monocytes according to several references and various online databanks^2^. It has also been detected in a variety of leukemic and lymphomatous cells, cancerous cell lines, and carcinomas as well as in myeloma cell^1^.

#### Cellular distribution

In non-epithelial cells, KCC1 acts mainly as a plasma membrane carrier system [11, 13]. Whether it could play a role in intracellular organelles has not been reported thus far. In epithelial cells, KCC1 also mainly acts at the cell surface but is confined to the basolateral membrane based on all accounts in the biomedical literature.

### Function and regulation of KCC1 in animal species

#### Transport characteristics

There is a paucity of data regarding the transport characteristics of KCC1 per se. While it is mainly through transport assays in RBC that the functional signature of K^+^-Cl^−^ cotransport was determined, it is now known that this transport function is accounted for by at least two KCC isoforms or splice variants in most cell types and tissues [15, 38–40]. Accordingly, K^+^-Cl^−^ cotransport in native environments cannot be ascribed to the sole activity of KCC1.

While this limitation should be kept in mind, the studies in RBC showed that K^+^-Cl^−^ cotransport was associated with the movement of one cation per one anion during each transport cycle [41] and that it was therefore outwardly directed. Of notice, however, the stoichiometry of ion transport by KCC1 per se has still not been confirmed experimentally and the number of ion binding sites for either of the Na^+^-independent family members has still not been determined [3, 4].

Through more recent work in heterologous expression systems, the affinity of KCC1 for the transported ions was found to be in the same range as that of KCC3 and KCC4 but its transport capacity to be relatively lower [11, 42, 43]. Interestingly, Bergeron et al. [43] further observed that K^+^ ions could be efficiently substituted for by NH_4_^+^ ions at the K^+^ translocation site of KCC1. For this reason, they came to the conclusion that this transporter could also be involved in acid uptake and had the potential to regulate both intracellular pH (pH_i_) and extracellular pH (pH_e_).

As for the effect of various agents on KCC1 activity per se, it was found to be as described in native cells types or tissues and similar among the isoforms. In essence, the pharmacological signature of KCC1 was characterized by the following traits: stimulation by *N*-ethylmaleimide, modest inhibition by bumetanide, DIDS, and barium, and stronger inhibition by furosemide and DIOA [11, 23, 24, 42, 43]. Importantly, several of these traits were observed in at least two heterologous expression systems.

#### Regulation

The N- and C-termini of KCC1 are both predicted to be cytosolically disposed, implying that they could interact with a number of signaling intermediates, cytoskeletal elements, and vesicle-associated membrane proteins. However, there are only a few consensus phosphoregulatory sites within these domains and most are for CK2 and PKC phosphorylation (Fig. 1a). In the C-terminus, there is also a leucine zipper domain and a paired amphipathic helix repeat that could sustain the assembly of KCC1 into CCC-based heterodimers [44] or its interaction with cytoskeletal elements [45].

Based on elegant studies by Rinehart et al. [46], cell swelling was found to activate KCC1 through dephosphorylation of residues T_926_ and T_983_ by a phosphatase type 1 (see Fig. 1a). In addition, the same residues were found to be phosphorylated under isotonic condition, i.e., when KCC1 is in its inactive state, through the WNK kinase/OSR1-dependent pathway. In subsequent studies, Frenette-Cotton et al. [47] have shown that additional Ser/Thr sites were probably at play given that cell swelling caused an overall increase in the phosphorylation state of another KCC isoform.

Otherwise, a number of studies have shown that K^+^-Cl^−^ cotransport could be affected through changes in intracellular O_2_ pressure (pO_2i_) and Mg^2+^ concentration (Mg^2+^_i_) as well as through the involvement of cytoskeletal elements. The importance of these factors in KCC1 regulation will be outlined below while discussing the pathophysiology of RBC dehydration in sickle cell anemia, a disease where Hb^α/α;β/β^ (HbA) is replaced by Hb^α/α;S/S^ (HbS) through sickling mutations in both of the β chains.

### Physiological roles of KCC1

#### Preamble

The physiological roles of KCC1 per se have not been studied as extensively as those of the other Na^+^-dependent CCC. There are probably at least three reasons as to why: (1) KCC1 might have been predicted to play the same role in many cell types by acting as a widely distributed housekeeping isoform. (2) KCC1 might have also been predicted to play a redundant role, especially in cell types where other Na^+^-dependent CCC with higher transport activity are expressed. (3) Over the years, KCC2 and KCC3 have generated most of the attention among the Na^+^-independent CCC because of their associations with neurological disorders [8–10].

Whether or not these reasons could have explained why KCC1 was subjected to more limited investigative efforts, they should be considered as unfounded as it stands. In particular, the characterization of mouse models inactivated for *Kcc1* has now provided evidence to suggest that this gene could play important and specific roles in various cell types including those of the hematopoietic lineage. A review of the data available will be summarized hereafter.

#### Role of KCC1 in RBC

RBC is known to express KCC1, KCC3, and KCC4 [15, 38–40]. Two of the isoforms present also come as two splice variants each. However, there is evidence to suggest that K^+^-Cl^−^ cotransport in this cell type could be accounted for by KCC3B predominantly. For instance, Pan et al. [39] have found that RTC and mature RBC from mouse and human expressed this variant protein at relatively comparable levels whereas RTC expressed KCC1 at much higher levels than mature RBC. Along the same line, Rust et al. [15] have found that *Kcc3*-null mouse RBC exhibited lower K^+^-Cl^−^ cotransport than *Kcc1*-null mouse RBC.

For various reasons, however, it is not clear that KCC3 would play such a preponderant role in mature RBC. First, KCC1 and KCC3 were not detected by Pan et al*.* [39] through the same antibody. If KCC1 was actually much more abundant than KCC3 in RTC, it could then be as abundant as, or even more abundant than KCC3 in mature RBC. Second, while the genetic background used by Rust et al. [15] was not the same for all of the models characterized, it is known to affect K^+^-Cl^−^ cotransport in RBC [48]. Third, KCC1 does appear to be involved in sickle cell anemia as will be discussed below.

The data of Pan et al*.* [39] are nonetheless consistent with the idea that KCC1 could play a role in erythroid maturation and are in keeping with those of two other research groups. Indeed, Pellegrino et al. [49] demonstrated that human or mouse HbA RTC only expressed full-length KCC1 transcripts during the early stages of cell differentiation, and Su et al. [12], that KCC1 expression was higher in enriched population of HbS RTC. Given that RTC and mature RBC are both endowed with K^+^-Cl^−^ cotransport activity [12, 39, 50, 51], the data of Pan et al. also suggest that some of the carrier isoforms could exhibit exceptionally slow turnover rates beyond the RTC stage.

As alluded to already, it is now widely accepted that K^+^-Cl^−^ cotransport at the RBC surface plays a central role in the pathophysiology of sickle cell anemia [14, 15]. In this inherited disorder, the abnormal hemoglobins expose a hydrophobic domain between the E and F helices when they are deoxygenated and polymerize with each other to form rigid precipitates that anchor themselves to the cytoskeleton [52, 53]. Sickling also comes with lower cell volumes due to an overactive K^+^-Cl^−^ cotransport function [54, 55] that contributes to cell rigidity by increasing viscosity and polymer concentration.

Rust et al. [15] have demonstrated the importance of this mechanism by studying a mouse model of *Kcc1* and *Kcc3* inactivation in the SAD transgenic background of hyper sickling human HbS. In particular, they found that RBC in *Kcc1*^−/−^*Kcc3*^−/−^_SAD_ mice was clearer and larger than in *Kcc1*^+/+^*Kcc3*^+/+^_SAD_ mice. However, inactivation of either carrier in the SAD background revealed that KCC3 played a more important role than KCC1 in sickle cell formation. Given that mature RBC can also acquire the abnormal phenotype, excessive K^+^-Cl^−^ cotransport could be contributed for by KCC1 beyond the RTC stage of erythroid differentiation, at least in the case of HbS cells.

Another group has demonstrated the role of KCC1 in sickle cell formation by studying a mouse model in which the transporter is constitutively activated through a phosphorylation-precluding mutation (M935K) in its C-terminus [14]. On its own, the *Kcc1*^*M935K/M935K*^ mouse model resulted in semi-dominant RBC microcytosis, and when bred into the humanized heterozygote HHb^α/α;β/S^ mouse model, in widespread sickling-induced tissue damage. As such, this group provided direct evidence that excessive K^+^-Cl^−^ cotransport did contribute to sickle cell formation and that it could affect the erythropoietic lineage beyond the RTC stage of differentiation.

Despite the importance of previous findings, the mechanisms of increased K^+^-Cl^−^ coefflux in sickle cell anemia are still largely elusive. A change in carrier abundance is probably partly at cause given expression levels of KCC1 and KCC3 are higher in HbS RTC than in HbA RTC and higher in mature HbS RBC that in mature HbA RBC [12, 49]. However, mature RBC does not have the capability of upregulating total protein expression, and they can also undergo sickling after only 2 h of hypoxia [56]. As such, dehydration of these cells by KCC1 would probably require unitary transport rates or capacity to increase as well.

One of the mechanisms that could account for the increase in K^+^-Cl^−^ coefflux pertains to the dependence of this transport moiety on pO_2i_ [57–61]. Even if K^+^-Cl^−^ coefflux and pO_2i_ are linearly interrelated in HbA cells and even if sickled cells form at low pO_2i_, there is evidence to suggest that hypoxia could still be a cause. Indeed, the relationship between K^+^-Cl^−^ coefflux and pO_2i_ in HbS cells is U-shaped instead of linear [57, 58, 61]. In the cytosol of sickled cells, additionally, the effect of hypoxia on O_2i_ availability could be attenuated by an abnormal Bohr effect [62]. However, K^+^-Cl^−^ coefflux in these cells is not much higher at very low pO_2i_ than it is at 100% pO_2i_ [63], and one would not expect the Bohr effect to counteract the effect of hypoxia completely.

How hypoxia could affect K^+^-Cl^−^ coefflux is in itself unknown. Some investigators have argued that low pO_2i_ could cause this carrier system to become more active in HbS cells by decreasing pH_i_ [63–65]. However, other investigators have shown that the activity of both KCC1 and KCC3 decreased progressively below pH_i_ levels of 7.0–7.1 and that the only isoform that could potentially increase its activity under such circumstances is KCC4 [43]. Thus far, however, the role of this other isoform in sickle cell anemia is controversial.

**Fig. 2:**
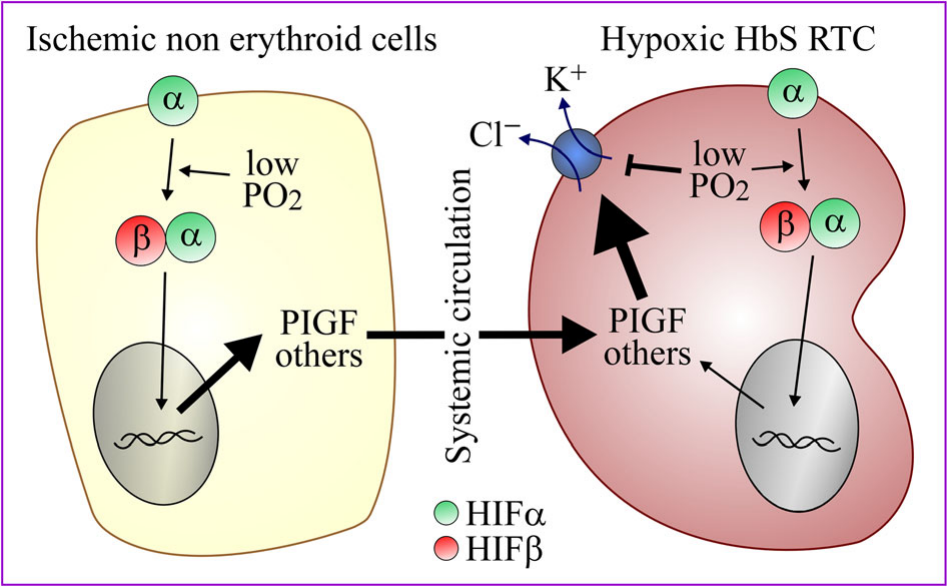
Regulation of K^+^-Cl^−^ cotransport in HbS cells. During occlusive crises, PGIF are produced from ischemic non-erythroid tissues and taken up by RTC where it could increase KCC1 expression and overcome the potential inhibitory effect of low pO_2i_ on K^+^-Cl^−^ cotransport. Abbreviations: HbS, hemoglobin S; HIF, hypoxia-induced factor; PIGF, placental growth factor

There is evidence to suggest that hypoxia might still explain why K^+^-Cl^−^ coefflux is increased in HbS cells as it could do so through systemic rather than local effects. Indeed, placental growth factor (PIGF) has been found at high circulating levels in sickle cell anemia, probably as a result of HIF1α upregulation in ischemic tissues, and to increase KCC1 expression in an erythroid RTC type cell line [66]. Given, however, that PIGF is also upregulated in normal RTC by low pO_2i_ [67], its synthesis by non-erythroid cells would have to be sufficiently important to bypass any inhibitory effects that low pO_2i_ might exert on KCC in HbS cells (Fig. 2). It would not be predicted to affect mature RBC either if its effect was to alter total KCC1 expression primarily.

The dependence of K^+^-Cl^−^ coefflux on Mg^2+^_i_ is another factor that could contribute to the transport phenotype of HbS cells. Indeed, while K^+^-Cl^−^ coefflux is known to be stimulated at lower Mg^2+^_i_, the cytosol of sickled RBC is also known to be Mg^2+^-deficient [59, 68, 69]. Once again, however, the mechanisms and isoforms involved have not been deciphered. Some investigators have suggested that low Mg^2+^_i_ could act by modulating the activity of signaling intermediates [63] and others by affecting the cytoskeleton (see below). Despite the unknowns, the sensitivity of K^+^-Cl^−^ coefflux to Mg^2+^_i_ is still of interest given that it has prompted clinical studies to determine the efficacy of Mg^2+^ supplementation in the treatment of sickling disorders [70].

A third mechanism could involve the disassembly of spectrin by HbS, i.e., of a cytoskeletal element that is normally formed of four subunits (α1/α1;β1/β1) and associates with the inner bilayer. This mechanism is suggested by two sets of deduced observations. The first one is that variety of intracellular inorganic cations have been found to inhibit K^+^-Cl^−^ cotransport and that they could do so by shielding negative charges at the surface of the inner bilayer [45, 71]. The second one is that a rise in temperature has also been found to stimulate K^+^-Cl^−^ cotransport [45, 72] and that it could do so by exposing negative charges from the same inner bilayer through spectrin disassembly [45].

As alluded to, regulatory enzymes are additional players that could contribute to upregulation of K^+^-Cl^−^ cotransport in HbS cells. They include the WNK kinases that come as four isoforms and are known to inhibit KCC1 and KCC3 [46, 47, 73–76]. If these enzymes did play a role, their activity should thus be reduced in HbS cells. In this regard, interestingly, D_368_ in rat WNK1 and D_293_ in WNK3 have been shown to act as binding sites for Mg^2+^ and their replacement by Ala residues to abolish the kinase activity of these enzymes [77, 78]. The inner bilayer is also an important component of the WNK kinase-dependent signaling pathway [45, 77–80].

Other ion transport pathways could play a role in the dehydration of HbS cells [81–87]. They include the mechanosensitive ion channel PIEZO1 (also called Psickle) and the Gardos channel (also called KCNN4). In particular, both these pathways are sensitive to pO_2i_ and are upregulated in sickled RBC [82, 88, 89]. In the past, inhibition of KCNN4 by clotrimazole and senicapoc has also been under clinical studies for the treatment of sickle cell anemia [87, 90, 91]. More recently, senicapoc has been renamed to PF-05416266 (Pfizer Inc., New York, NY, USA) and has become the object of a new trial for the same indication.

#### Cancer

At least three members of the KCC family have been shown to affect cancer cell proliferation, growth, and invasiveness. The mechanisms involved are still unknown but could implicate various effectors that are sensitive to changes in intracellular Cl^−^ concentration (Cl^−^_i_), cell volume, or membrane potential. Alternatively, cancerous cell transformation could cause KCC activity to be affected secondarily through concomitant changes in pO_2i_, cell volume, signaling activity, cytoskeletal organization, and transcription efficiency.

As for KCC1 more specifically, it is suspected of facilitating growth and invasiveness for cervical and endometrial adenocarcinomas. In the presence of IGF, for instance, cell lines derived from such cancers have been found in some studies to exhibit increased ERK-dependent signaling and invasiveness, but not so if KCC1 activity was inhibited concomitantly through pharmacological agents or RNA interference [16, 92]. In these studies, KCC1 expression was also stimulated by IGF, but according to other accounts, it is typically low in many types of cancer cells, undetectable in lymphoma cells, and positively correlated with higher survival rates in renal cell carcinoma^1^.

We postulate that the HIF-dependent pathway could play an important role in regulating KCC1 expression at the surface of cancer cells. In particular, this pathway could be activated through somatic mutations in the VHL gene or through low pO_2i_ levels as cancer cells proliferate into solid masses. The involvement of HIF under such circumstances could then explain why higher KCC1 expression is associated with a better prognosis in renal cell carcinomas as it would then point towards the presence of pathogenic modifications in the VHL gene [93, 94].

#### Bone

Kajiya et al. [18] have found that KCC1 was expressed in mouse osteoclasts based on reverse transcriptase (RT)-PCR measurements, immunohistochemical studies, and western blot analyses. Surprisingly, they found that KCC2 was also expressed in this cell type, albeit at much lower levels, and that KCC3 and KCC4 were undetectable. Transcript abundance inferred from the EST databanks is partly consistent with such findings^1^. In human and mouse bone, there are indeed 55 and 410 messages per million (MPM) for KCC1, respectively, and 0 MPM for KCC2 and KCC4. In human bone, however, there are also 97 MPM for KCC3, suggesting that this isoform is expressed in other cell types such as osteoblasts and osteocytes.

**Fig. 3:**
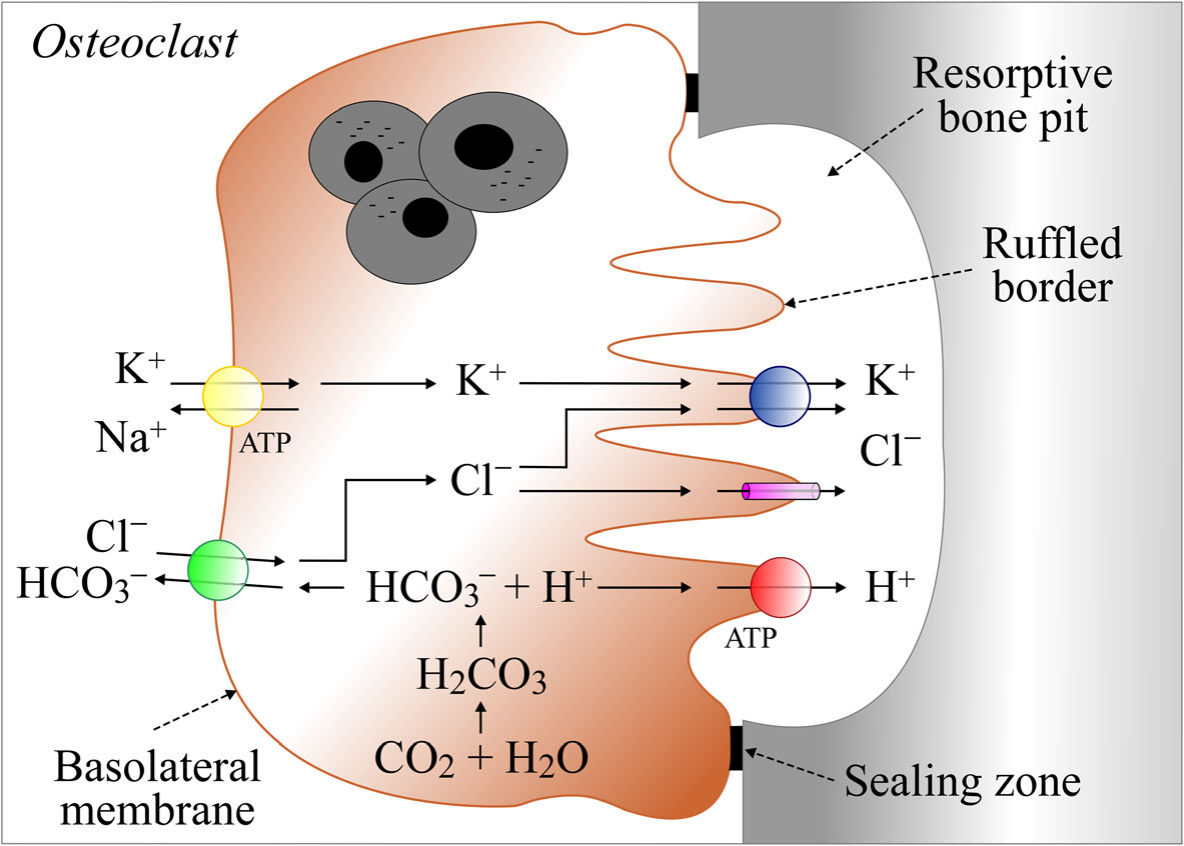
Role of KCC1 in osteoclasts**.** On the ruffled border, transport systems shown consist of KCC1, the Cl^−^ channel CLC-7 [95], and the vacuolar H^+^-ATPase pump ATP6V1C1 [96]. On the basolateral membrane, they consist of the Na^+^/K^+^-ATPase pump ATPA1B1 [97] and the Cl^−^/HCO_3_^−^ exchanger SLC4A2 [98]. On the ruffled border, the role of KCC1 could be to use the K^+^ gradient generated by the Na^+^ pump to provide an accessory route for Cl^−^ secretion in resorptive pits [18]. If, alternatively, KCC1 was localized on the basolateral side, it could then serve two purposes. The first one would be to sustain Cl^−^/HCO_3_^−^ exchange by providing the antiporter with a continued supply of Cl^−^ ions. The presence of KCC1 at this location would thus allow secondarily for higher H^+^_i_ and luminal H^+^ secretion. The second one would be to sustain Na^+^/K^+^-ATPase activity by providing the enzyme with a continued supplied of K^+^ ions. The presence of KCC1 at this location would thus allow secondarily for higher intracellular negativity and luminal Cl^−^ secretion

In the same study by Kajiya et al., KCC1-specific antisense oligonucleotides were also shown to suppress pit formation in calcified bone, and DIOA to increase Cl^−^_i_ and H^+^_i_ in osteoclasts. Although it was not clear in this work that KCC1 was expressed in the ruffled border of osteoclasts, Kajiya et al. suggested that at this location, KCC1 could provide an extrusion mechanism for Cl^−^ during bone resorption to facilitate H^+^ secretion in forming pits (see Fig. 3). Interestingly, other studies have shown that the Cl^−^ channel CIC7 also provided an extrusion mechanism for Cl^−^ and that mice inactivated for the encoding gene exhibited severe osteopetrosis. Whether the same skeletal phenotype would be observed in *Kcc1*-null mouse does not appear to have been reported as of yet.

NCC, another member of the CCC family, has been drawing attention in the field of osteoporosis for many years because its inactivation—through thiazide therapy or homozygous loss of function mutations—has been shown to increase bone mineral density [99]. While this effect has been generally attributed to the secondary role of NCC in Ca^2+^ handling by the gut and the kidney [100, 101]—NCC inactivation increases Ca^2+^ absorption in both epithelia—it could also be attributed to the presence of NCC in the bone. In particular, this CCC was shown to be much less abundant in differentiating than in proliferating human and fetal rat osteoblasts, and its inactivation to increase bone mineralization and expression of osteoblastic differentiation markers [102].

Taken together, the findings described in this section of the review highlight the potential importance of Cl^−^_i_ or of cell surface Cl^−^ transport on bone cell function. Higher Cl^−^_i_ or lower Cl^−^ efflux in osteoblasts (as would occur through decreased KCC1 activity) appears to be associated with decreased bone resorption, whereas higher Cl^−^_i_ or lower Cl^−^ efflux in osteoclasts (as would occur through increased NCC activity or decreased KCC3 activity) to be associated with decreased bone formation.

## Conclusion

This review has allowed to show that KCC1 accomplishes specific physiological and pathophysiological roles in animal cells and that it does not act solely as a housekeeping K^+^-Cl^−^ cotransport mechanism. As it stands, however, it is mainly in RBC maturation and sickling of RBC that such roles have been demonstrated more convincingly. There is still emerging evidence to suggest that KCC1 is also of functional relevance in cancer development and in bone resorption.

As mentioned, KCC1 is ubiquitously expressed and could thus play roles in many other cell types within the hematopoietic lineage. In this regard, KCC3 has been found to sustain hypochlorite synthesis by white blood cells through its Cl^−^ cotransporter function in phagosomes [103, 104]. Along the same line, it is particularly intriguing that KCC1 is expressed in a variety of leukemic cells but that it is virtually absent from most types of lymphoma cells. It is thus tempting to postulate that the chromosomal locus of KCC1 (16q22.1), which is known to harbor cancer-associated genes such as CDH1 and CDH3, is altered in these cells through DNA rearrangements [105]^1^. Alternatively, low K^+^-Cl^−^ cotransport activity could confer a survival benefit to a variety of lymphoma cells.

It is perhaps also intriguing that there are still no reports of human disorders that have been linked to pathogenic mutations in *Kcc1*. As suggested by the mouse models, the reason may be that this gene plays a redundant role and that its inactivation is thus tolerated under normal condition. If and when disease-causing mutations are identified, KCC1 will certainly find a place of honor among the other family members. The same will also be true if pharmacologic inactivation of this isoform in sickle cell anemia were to improve the morbidity and mortality that is associated with this prevalent disorder.

## Data Availability

All data generated or analyzed during this study are included in this published article (see Footnotes).

## Footnotes

1 Sequences used to generate the phylogram of Fig. 1b:

- CCC Accession numbers
- NKCC1 NP_001037.1
- NKCC2 NP_000329.2
- NCC NP_000330.2
- KCC1 NP_005063.1
- KCC2 NP_001128243.1
- KCC3 NP_598408.1
- KCC4 NP_006589.2
- CCC8 NP_064631.2
- CCC9 NP_078904.3

2 Web links exploited:

- https://www.ncbi.nlm.nih.gov/geoprofiles/?term=SLC12A4;
- https://www.ncbi.nlm.nih.gov/unigene/?term=SLC12a4;
- https://www.proteinatlas.org/ENSG00000124067-SLC12A4/pathology/tissue/renal+cancer.
